# Impact of screening on cervical cancer incidence and mortality in a Northern Brazilian city

**DOI:** 10.3332/ecancer.2022.1418

**Published:** 2022-07-01

**Authors:** Lucrecia Aline Cabral Formigosa, Luciana Ferreira dos Santos, Joana Dulce Cabral Formigosa, Denise da Silva Pinto, Danielle Saraiva Tuma dos Reis, Marcos Valério Santos da Silva

**Affiliations:** 1 Federal University of Pará (UFPA), Belém, Pará, 66050160, Brazil; 2 Population Based Cancer Registry of Belém, Belém, Pará, Brazil

**Keywords:** cervical neoplasm, incidence, mortality, cancer registry

## Abstract

**Objective:**

To analyse the impact of screening actions on the incidence and mortality rates of cervical cancer (CC) in the city of Belém, Brazil.

**Methods:**

Based on the cancer registry data from 1998 to 2017, collected from the Belém Population-Based Cancer Registry, combined with local population data for the interval 1998–2017, CC incidence and mortality were calculated. The Segi world population 1960 was used for age-standardised incidence/mortality rates.

**Results:**

In the period analysed, there were 4,469 new cases and 1,660 deaths from CC. The median age at diagnosis of invasive cases was 51 years. The age-adjusted incidence rate decreased from 18.65/100,000 in 1998 to 11.79/100,000 in 2017, despite the increase observed in the first 5 years of the historical series, while there was stability in mortality rates in the same time lapse.

**Conclusion:**

CC is still one of the most common malignant tumours that threaten public health in northern Brazil. The trend of the disease depends on comprehensive prevention and control strategies regarding the local situation and age groups, with emphasis on the organisation of the screening programme and vaccination against human papillomavirus.

## Introduction

Cervical cancer (CC) is the second most prevalent cancer in women after breast cancer. It is the fourth leading cause of cancer mortality in the country and the mortality rate is higher in black women and women residing in the northern, southern and central-western regions of Brazil [1]. In addition, each region has specific traits regarding race and marital status. White women who died had some form of stable union during their lifetime, while other races are more associated with single marital status [2].

Worldwide, CC is the fourth most common type of cancer diagnosed among women [3]. Morbidity and mortality from this neoplasm are lower in developed countries due to the availability of efficient and affordable screening programmes and diagnostic and treatment facilities. Meanwhile, in low- and middle-income countries, the low survival rate is attributable to late diagnosis and failure to receive or complete prescribed treatment regimens among patients [4].

Over the past four decades, the incidence and mortality rates for CC have decreased due to population-based screening programmes and human papillomavirus (HPV) vaccination [5], as occurred in developed countries where the reduction reached levels of up to 80% [6].

In Brazil, numerous initiatives have been undertaken at the national level over the past decades, including the *Viva Mulher* Programme in 1998, the National Policy for Oncological Care in 2005 and, more recently, the National Policy for Cancer Prevention and Control in 2013. All of them aimed at the organisation of the line of care, with emphasis on screening and treatment of precursor lesions [7].

Despite the relevance, even today there are few studies that analyse the individual–environment relationship with regard to the neoplasm, although regional variations in the incidence rates of CC point to the causal role of regional, social and environmental aspects of each population [8]. Moreover, regional differences also influence treatment opportunities and survival of women with CC [9], hence the importance of developing research in the Brazilian Amazon region, where the disease has great epidemiological impact.

From this context, the study aimed to analyse the impact of screening actions on the incidence and mortality rates related to CC in the city of Belém, Brazil, between the years 1998 and 2017.

## Methods

This is an ecological time-series study. The study population was composed of women living in the municipality of Belém, capital of the State of Pará, located in the northern region of Brazil. The city had an estimated population of just over 1.5 million inhabitants in the year 2021, of which 750,000 were women [10].

The incidence data were extracted from the Population-Based Cancer Registry of Belém (PBCR Belém), while the mortality data were extracted from the Mortality Information System, both available at the electronic address of the Department of Information and Informatics (DATASUS, http://www2.datasus.gov.br/DATASUS.index.php).

The PBCR Belém collects information from public and private hospitals and laboratories in the coverage region, in addition to including information from other Brazilian health information systems, especially those related to cancer screening, according to recommendations from the National Cancer Institute of Brazil [11] and the International Agency for Research on Cancer [12].

Cases available in the databases from 1998 to 2017 were included, which were selected because they were the most recent at the time of data collection. All tumours coded as C53 by the International Classification of Diseases, 10th Revision. Data access occurred in May 2021.

Crude incidence and mortality rates, expressed per 100,000 women, were calculated using Belém’s estimated and projected mid-year population from the latest 2010 Brazilian census as the denominator. Age-adjusted rates were calculated by the direct method with the 1960 standardised world population [13].

Time trends in incidence and mortality rates were calculated using Joinpoint Regression software, version 4.8.0.1, Bethesda, MD, USA [14], using the simple linear regression to identify time change points and estimate the annual percentage change (APC%), assuming that the rates change with a constant percentage on a logarithmic scale for each time segment.

This study analysed the available and open secondary data. The data are disclosed with unrestricted use and access, not being required, for this study, evaluation of the Research Ethics Committee, under the terms of the Resolution of the National Health Council No. 466, 12 December 2012.

## Results

From 1998 to 2017, 4,469 new cases and 1,660 deaths from CC were recorded. The median age at diagnosis of invasive cases was 51 years. The age-adjusted incidence rate decreased from 19.26/100,000 in 1999 to 11.72/100,000 in 2017. Despite the increase seen in the first 5-year time series, there was a statistically significant decrease in age-standardised rates (ASR) for incidences starting in 2003. The mortality figures in the region showed fluctuation over the two decades analysed, tending towards stability.

**Figure 1.**
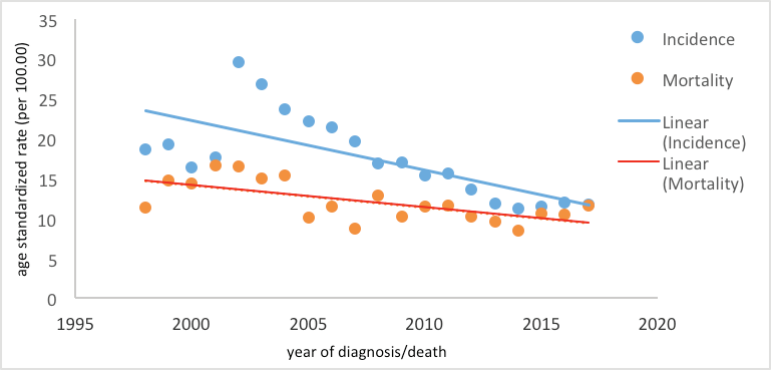
Trends in CC incidence and mortality in Belém, Brazil, from 1998 to 2017.

Table 1 and Figure 1 show the number of cases, crude rates and ASR for the incidence and mortality of CC seen annually in Belém from 1998 to 2017.

**Figure 2.**
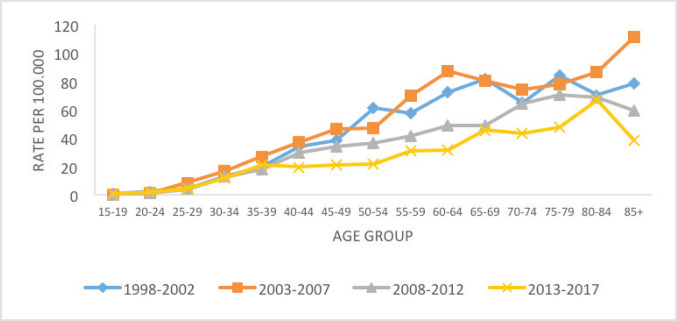
Age-specific incidence of CC for the 5-year period, Belém, Brazil, from 1998 to 2017.

In all periods, it is observed that the age-specific rate of incidence by CC increases after 25 years of age. The decrease in rates was evident for all ages; however, the pattern of variation of this indicator is different according to age group, with a greater reduction in incidence in the age group over 60 years in the period from 2008 to 2017, and less effect below 40 years (Figure 2).

**Figure 3.**
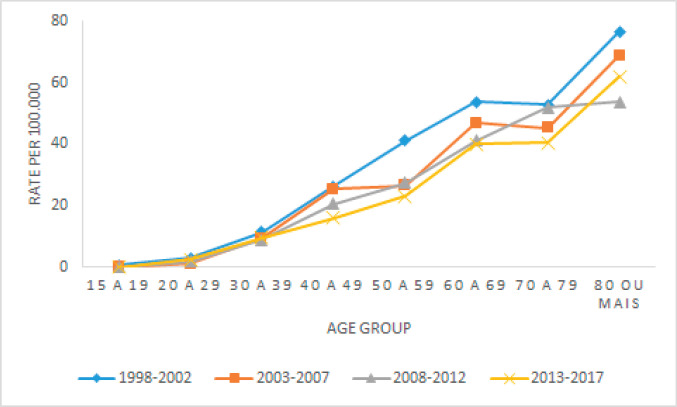
Adjusted CC mortality rates for the 5-year period, Belém, Brazil, from 1998 to 2017.

In relation to the adjusted mortality rates for CC according to age group in Belém (Figure 3), an increase in the number of deaths is identified in all periods according to the aging of the population, more accentuated above 50 years of age, when the ASR is almost double in relation to women between 40 and 49 years of age.

## Discussion

In the course of the 20 years analysed, the reduction in the incidence rates of CC in the region was evident, following the trend observed worldwide in the first decade of the 21st century, moving from third to fourth place among the most frequent neoplasms, excluding non-melanoma skin cancers [15].

The significant reduction of the disease in these countries can be explained by the Institution of National Programmes of early detection, through conventional cytology or HPV testing in earlier years, including the population discovered by the health system, as occurred in the United States [16, 17].

In the locality of the study, the favourable trend of reduced incidence can be attributed to the positive results of government programmes implemented for prevention and control of the disease. The first of them, *Viva Mulher* programme, started in 1998, aimed at standardising the collection of the cytology cervical tests (Pap smear), through the training of primary healthcare professionals [18].

In 2005, the National Policy for Oncological Care was instituted, replaced in 2013 by the National Policy for Cancer Prevention and Control, with a view to organising the line of care and the network of health services and emphasising integrality and health information. In addition, for the monitoring and management of actions, they encouraged the implementation of population-based and hospital-based cancer registries [19].

Other cross-cutting policies have also included the topic of CC as a goal, among them are the Strategic Action Plan for Confronting Chronic Non-Transmissible Diseases (2011–2020), Pact for Life, National Policy for Basic Care, Policy for Integral Care of Women’s Health and National Policy on STD/AIDS [7]. HPV vaccination was included in the National Immunisation Programme in 2014 for girls and boys from 9 and 11 years of age, respectively. In the same year, the *Qualicito* Programme was created to improve the sensitivity of the cytology test [20–22].

It can be identified that ASR in Belem (11.72/100,000 in 2017) is far below the regional and national estimates, which were 29.05/100,000 (26.00–33.04) and 18.39/100,000 (17.63–19.17), respectively, both calculated from the Global Burden Disease study for the year 2017 [23].

Among other factors, geographical variations are usually associated with socio-economic conditions, represented by the Human Development Index (HDI), resulting in incidence rates ranging from 3 to 70 cases per 100,000 women worldwide [24]. Brazil, due to its large territorial dimensions, presents very marked disparities in HDI when comparing the various regions, which provides different disease profiles [25]. In this sense, the HDI of the municipality of Belém is equivalent to 0.746, while in the state of Pará it is 0.5786 [26].

The progressive increase in ASR observed in the first 5-year period of the series is explained by the beginning of the pilot screening project in 1996, in Belém, expanded later in 1998, when a greater number of cases were diagnosed [18]. This increase is followed in 2003 by a drop in the registered values, an effect of the identification of incident and prevalent cases in the first years of the screening programme, continued by the subsequent identification of only incident cases in the programme [12].

The age-specific analysis clearly indicates that CC occurred at various ages, during which adult women have many economic and care responsibilities for their families [24]. In all periods of this study, the peak of the disease occurred in women of senile age (over 60 years), possibly due to the peculiarities of the population, age-related changes in anatomy and the periodic reactivation of HPV infection, with delayed reactivation [17].

It was also evident that mortality had a greater impact on older women compared to younger women. This may reflect exposure to risk factors and inequalities in access to adequate screening and timely treatment [27]. It is noteworthy that the occurrence of CC in elderly women depends on adherence to screening between 50 and 60 years of age, as well as on the effectiveness of the examination despite the anatomical changes that occur with advancing age, as reported in the previous paragraph [15].

The period analysed showed a stationary evolution of mortality rates, probably justified by easier access to early diagnosis of precursor lesions, at the same time that there was an expansion of cancer services in the city, with logistical and technological reorganisation of the care network, due to the implementation of the policies mentioned above [7].

It is important to clarify that, in Belém, the screening programme, by means of the Pap smear, has an opportunistic character, for being developed in the context of the demand for gynaecological care, prenatal care or family planning, which contributes to the increase in mortality, especially in this age group of the population [28].

Brazil still has high rates of cancers associated with infections, a profile of developing countries and a consequence of existing regional inequalities, ranging from differences in life expectancy, socio-economic conditions and access to health services for timely diagnosis and adequate treatment [29].

In addition, it was noted that the main risk factors for the development of the disease were having early sexual intercourse, low education, multi-parity, promiscuity, smoking, continuous use of contraceptive pills and, especially, HPV infection [30].

In this context, it is worth noting that the number of seniors who acquire sexually transmitted infections has increased in several countries around the world, especially those in development, due to lack of knowledge, unsafe sexual practice and the taboo that encourages social prejudice related to sexuality in the elderly, which ultimately make this population vulnerable to diseases [31].

Given the above, the late diagnosis of the disease also contributes to mortality from cancer, because in most cases, the difficulty of access to health services shows that there is a lack of quality in oncology services, especially in the regions surrounding large capitals, due to factors associated with low professional training in oncology care, the inability of health units to absorb the demand and difficulties of municipal and state managers to organise the levels of care [32].

Health education carried out in primary care is essential for the prevention and control of CC; however, opportunistic screening programmes, such as the one developed in the region, depend on the level of women’s knowledge about the disease and understanding of the importance of primary and secondary prevention, so that they can effectively contribute to reducing the rates of CC, as has occurred in previous years in Austria and France [33].

Therefore, it is necessary to design strategies directed towards older women, in order to increase the participation of this population in CC screening, since they are the most affected by diagnosis and death as a result of this cancer.

## Conclusion

This is the first study to analyse the incidence rates of CC in Belem, Brazil, based on data provided by the Cancer Registry.

Over the 20 years considered, consistent evidences were obtained on the effectiveness of the three distinct programmes implemented sequentially for cancer control since 1998. Soon after the introduction of screening programmes, CC incidence and mortality began to decrease, especially in young women, demonstrated by a simple temporal correlation cause–effect link. The time lag between the introduction of the programme and the significant rates decrease was very short.

The essential changes in the incidence and mortality of CC presented in this study seem to be consistent with the general health system and the opportunistic characteristics of the screening programme developed in the region. Thus, it is necessary to organise the actions of primary healthcare in order to improve early diagnosis, essential to improving the quality of life and increasing the survival of patients.

## Conflicts of interest

The authors declare that they have no conflict of interests.

## Funding

The authors received no specific funding for this work.

## Figures and Tables

**Table 1.** Cases, crude and ASR and APC, for CC incidence and mortality annually, in Belém, from 1998 to 2017.

	Incidence					Mortality			
**Year**	**Cases**	**Crude**	**ASR**	**APC**		**Cases**	**Crude**	**ASR**	**APC**
1998	160	13.63	18.65	0%		55	8.89	11.38	0%
1999	171	14.41	19.26	3.27%		74	11.83	14.74	29.53%
2000	165	12.96	16.30	14.85%		81	12.05	14.38	−2.44%
2001	191	14.44	17.67	7.38%		99	14.36	16.62	15.58%
2002	323	24.14	29.58	67.97%		97	13.98	16.48	−0.84%
2003	311	23.09	26.75	−9.57%		89	12.74	14.94	−9.34%
2004	273	20.14	23.68	11.48%		96	13.65	15.31	2.48%
2005	257	18.85	22.13	−6.55%		67	9.46	10.05	34.36%
2006	289	21.06	21.34	−3.57%		79	11.09	11.49	14.33%
2007	257	18.62	19.64	−7.97%		58	8.09	8.73	24.02%
2008	223	16.06	16.94	13.75%		90	12.46	12.81	46.74%
2009	241	17.26	17.03	0.53%		74	10.18	10.2	20.37%
2010	236	16.94	15.37	−9.75%		89	12.12	11.42	11.96%
2011	239	16.93	15.68	2.02%		91	12.37	11.64	1.93%
2012	207	14.59	13.64	13.01%		82	11.08	10.27	11.77%
2013	179	12.55	11.92	12.61%		76	10.21	9.61	−6.43%
2014	181	12.54	11.36	−5.79%		73	9.78	8.47	11.86%
2015	179	12.69	11.28	2.14%		94	12.52	10.65	25.74%
2016	195	13.60	11.98	4.45%		95	12.65	10.49	−1.50%
2017	190	13.32	11.72	−1.59%		101	13.45	11.55	10.10%
